# Accurate, automated classification of radiographic knee osteoarthritis severity using a novel method of deep learning: Plug-in modules

**DOI:** 10.1186/s43019-024-00228-3

**Published:** 2024-08-13

**Authors:** Do Weon Lee, Dae Seok Song, Hyuk-Soo Han, Du Hyun Ro

**Affiliations:** 1 Department of Orthopedic Surgery, Seoul National University College of Medicine, Seoul, South Korea; 2 CONNECTEVE Co., Ltd., Seoul, South Korea; 3 Department of Orthopedic Surgery, Seoul National University Hospital, 101 Daehak-Ro, Jongno-Gu, Seoul, 110-744 South Korea; 4 Innovative Medical Technology Research Institute, Seoul National University Hospital, Seoul, South Korea; 5 Department of Orthopedic Surgery, Dongguk University Ilsan Hospital, Goyang, South Korea

**Keywords:** Knee osteoarthritis, Deep learning, Classification

## Abstract

**Background:**

Fine-grained classification deals with data with a large degree of similarity, such as cat or bird species, and similarly, knee osteoarthritis severity classification [Kellgren–Lawrence (KL) grading] is one such fine-grained classification task. Recently, a plug-in module (PIM) that can be integrated into convolutional neural-network-based or transformer-based networks has been shown to provide strong discriminative regions for fine-grained classification, with results that outperformed the previous deep learning models. PIM utilizes each pixel of an image as an independent feature and can subsequently better classify images with minor differences. It was hypothesized that, as a fine-grained classification task, knee osteoarthritis severity may be classified well using PIMs. The aim of the study was to develop this automated knee osteoarthritis classification model.

**Methods:**

A deep learning model that classifies knee osteoarthritis severity of a radiograph was developed utilizing PIMs. A retrospective analysis on prospectively collected data was performed. The model was trained and developed using the Osteoarthritis Initiative dataset and was subsequently tested on an independent dataset, the Multicenter Osteoarthritis Study (test set size: 17,040). The final deep learning model was designed through an ensemble of four different PIMs.

**Results:**

The accuracy was the lowest for KL grade 1 (43%) and the highest for KL grade 4 (96%).

**Conclusions:**

The ensemble of PIMs could classify knee osteoarthritis severity using simple radiographs with a fine accuracy. Although improvements will be needed in the future, the model has been proven to have the potential to be clinically useful.

## Background

Kellgren–Lawrence grade (KLG) is one of the most commonly used criteria in classifying the severity of knee osteoarthritis (KOA) on a simple radiograph [1]. It categorizes KOA severity from grade 0–4 on the basis of joint space narrowing, osteophyte formation, subchondral sclerosis, and bony deformity observed in a simple radiograph of the knee. Despite its apparent simplicity, KLG varies even between expert surgeons or radiologists [2]. This is because the classification system is not a quantitative system and thus is often confusing, especially when diagnosed by clinicians with less experience in the field. For this reason, it would be useful to develop an accurate, automated prediction model of KLG.

During the past few years, there have been several efforts [3–10] to automatically classify radiographic severity of a knee with the aid of convolutional neural network (CNN), and the results have been promising. Deep learning (DL) methods are commonly used in this automatic KLG grading task since large scale data are utilized to improve model accuracy. However, these models are not flawless, including utilization of data with relatively low accuracy and low quality or volume. The accuracy levels were especially low when it came to discerning lower grades such as Kellgren–Lawrence grade [1] (KLG) 0 or 1. The accuracy of KLG 1 was only 11%, although the overall accuracy was 67% in a recent study [6]. This may be due to the fact that KLG 1 has less distinctive features than other higher grades because of the definition of “doubtful joint space narrowing and possible osteophytic lipping.” Therefore, the authors felt the need to design a new model that better predicts the KLG of a knee image using a more robust and objective dataset.

Recently, a plug-in module (PIM) [11] that can be integrated to CNN-based or transformer-based networks has been proposed to provide strongly discriminative regions for fine-grained classification, and the results have outperformed those of previous DL methods. Fine-grained classification deals with data with a large degree of similarity, such as cat species or bird species, and similarly, KLG image classification is one such fine-grained classification task. PIM utilizes each pixel of an image as an independent feature and can subsequently better classify images with minor differences. Therefore, the authors hypothesized that applying PIM in this task (KLG classification) might outperform the previous CNN-based models, especially in discerning lower grades of KLG that have less distinctive features.

The authors hypothesized that, as a fine-grained classification tasks, knee osteoarthritis severity may be classified well through the application of PIMs. Therefore, the purpose of this study was to develop a prediction model that automatically assesses the KLG of a knee image applying PIM. The authors tried to develop a model with better accuracy and better generalization in classifying the KLG of a knee than the models provided in the previous literature [3–10].

## Materials and methods

### Data composition

A retrospective analysis on prospectively collected data was performed. The dataset used for the study was a combination of two different open source datasets, the Osteoarthritis Initiative (OAI) [12] and Multicenter Osteoarthritis Study (MOST) [13]. OAI, which is funded by the National Institutes of Health, National Institute on Aging, and National Institute of Arthritis and Musculoskeletal and Skin Diseases, holds clinical data and X-ray images from 4796 individuals (41.5% men and 58.5% women) aged between 45 and 79 years. MOST is a similar project on osteoarthritis and holds data from 3026 individuals (60% men and 40% women) aged between 50 and 79 years old, although it has been recently closed to the public due to financial reasons. The details about the acquisition and protocols in the OAI and MOST studies are available online at https://nda.nih.gov/oai and http://most.ucsf.edu, respectively.

**Fig. 1:**
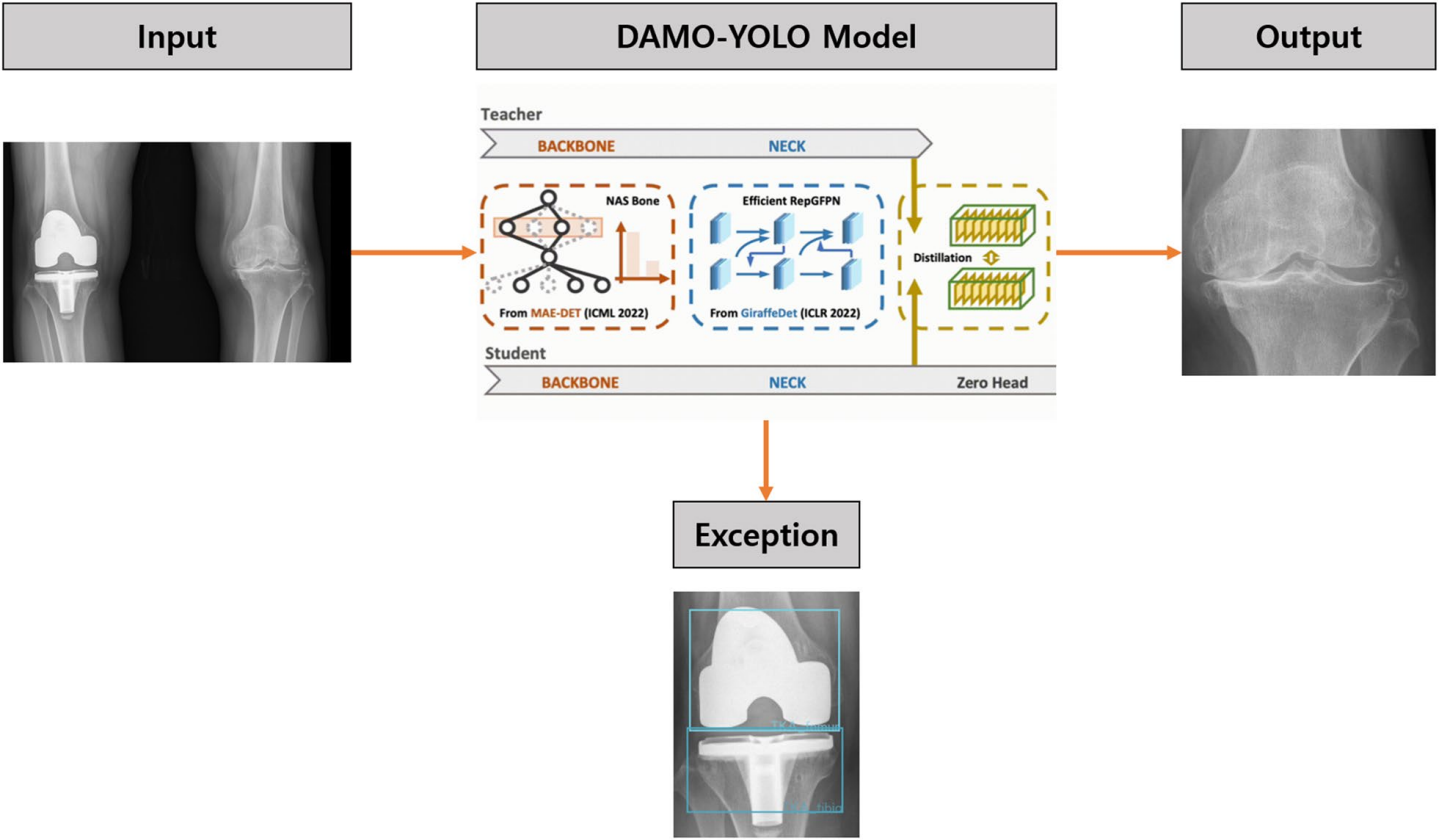
Knee localization process of an image in this study

The authors included all available knee radiographs from these two projects except for the images that did not have KLG labels. Subsequently, all images were cropped by range of interest (ROI) to include only one knee per image using a detection model (DAMO-YOLO [14]; Fig. 1). During the training process, 1439 images were used as training data, and the images were annotated either as a ROI of a knee image or of a metal implant in YOLO format. A single DAMO-YOLO model was developed to detect knee images while simultaneously ruling out the knees with metal implants. This detection model failed in only about 30 cases in which image contrast was significantly poor due to morbid obesity or inadequate radiograph filming. Finally, 63,688 images were selected, of which 46,648 images were used for training (37,462 images) and validation (9186 images) sets, and the remaining 17,040 images were used for the test set. The proportion of the training set:validation set was 8:2. The images from the MOST dataset were used as the test set, while training and validation sets were randomly assigned from the OAI dataset. Training and validation sets were divided so that the images of each set were from different patients, meaning that images of an individual patient are either in the training or validation set and not divided into both sets. This method was used to minimize the individual information unrelated to KLG that can be incorporated during model development.

### Image preprocessing and augmentation

Image resizing was initially performed to 384 × 384 pixels and 448 × 448 pixels each for two different backbone models of PIM [11], Swin [15] and EfficientNet [16], respectively. Horizontal flipping was utilized, and two images were provided per test image: the original test image and horizontally flipped test image. Then, image quality augmentation [17] was performed using random brightness, sharpening, image compression, Gaussian noise, histogram equalization and contrast limited adaptive histogram equalization [18]. Lastly, grayscale values of each pixel of an image were normalized according to the average and standard deviation value of the whole pixels of all images used in the study (mean = 0.543, standard deviation = 0.203) [19].

### Data labeling

Data labeling was performed using a vector for each KL (Kellgren–Lawrence) grade. The vector was designed also to either include one upper (or lower) grade to the initial KL grade to design a model that overestimates (or underestimates) the KL grade. This method, which has been first introduced as “label smoothing” in a previous literature [20], was attempted because prior KL grade models [3–6, 8, 9] and our trials showed that the models tend to confuse grade 1 and grade 2. Moreover, in a real clinical situation, even experts in this field sometimes overestimate or underestimate KLGs, and the authors tried to calibrate this. Several values were tried from 0.5 to 1 for the upper (or lower) grade labeling, and the vector that used 0.95 for the upper (or lower) grade labeling showed the most consistent accuracy for all grades. Therefore, each KL grade was labeled as shown below using this method.

**Table:** 

Upper-grade labeling	Lower-grade labeling
Grade 0 → [1, 0.95, 0, 0, 0]	Grade 0 → [1, 0, 0, 0, 0]
Grade 1 → [0, 1, 0.95, 0, 0]	Grade 1 → [0.95, 1, 0, 0, 0]
Grade 2 → [0, 0, 1, 0.95, 0]	Grade 2 → [0, 0.95, 1, 0, 0]
Grade 3 → [0, 0, 0, 1, 0.95]	Grade 3 → [0, 0, 0.95, 1, 0]
Grade 4 → [0, 0, 0, 0, 1]	Grade 4 → [0, 0, 0, 0.95, 1]

### Final modeling

**Fig. 2:**
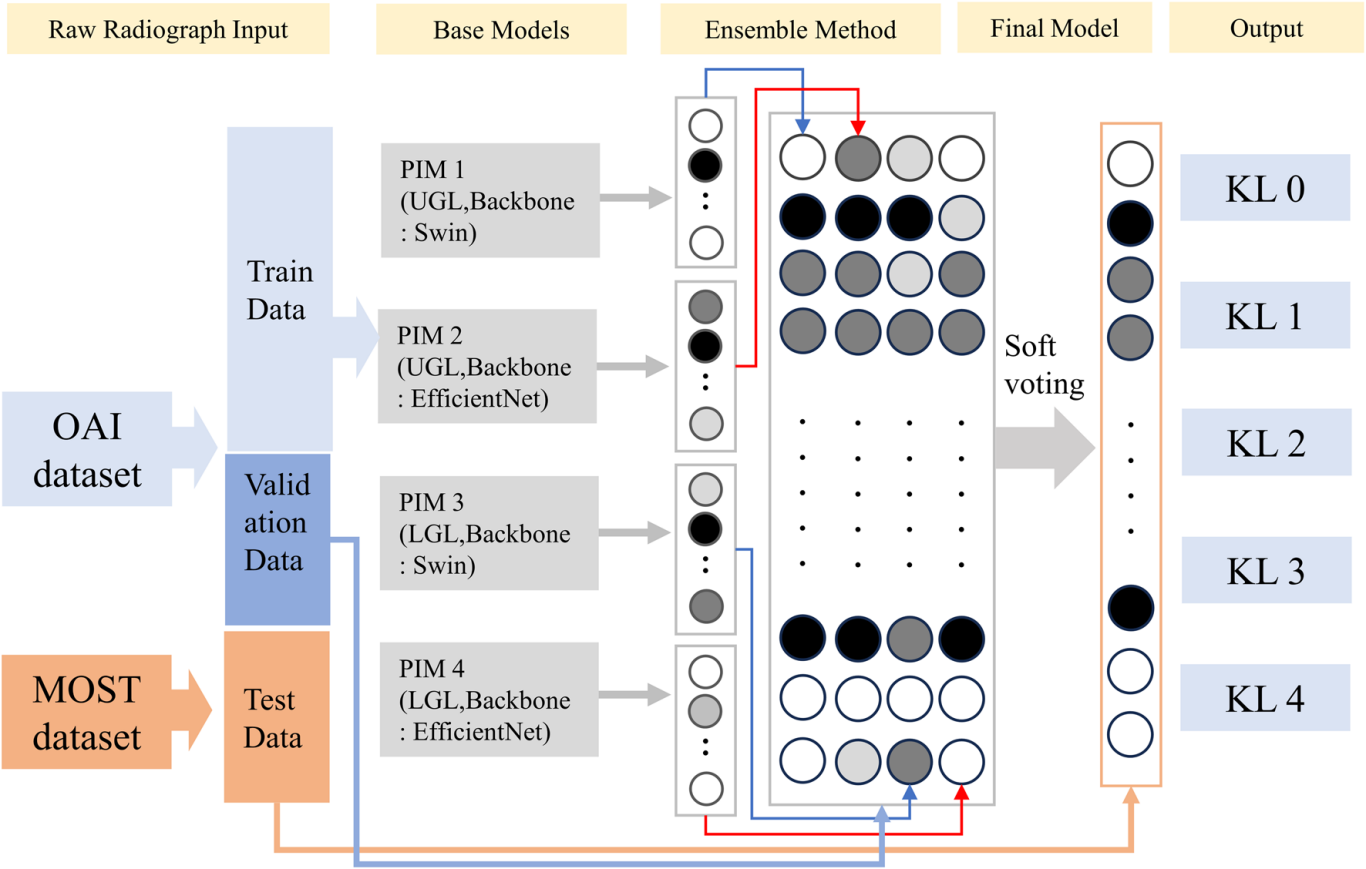
The overall architecture of model development in this study; OAI, Osteoarthritis Initiative; MOST, Multicenter Osteoarthritis Study; PIM, plug-in module; UGL, upper-grade labeling; LGL, lower-grade labeling; KL, Kellgren–Lawrence

The final DL model was an ensemble of four different PIMs that used Swin [15] and EfficientNet [16] as the backbone models (two each). Soft voting [21], which uses the probabilities of each model to infer the value, was chosen as the ensemble method. Two of the models applied the upper-grade labeling method, one of which used Swin and the other EfficientNet as the backbone model. The other two models applied the lower-grade-labeling method instead, one of which used Swin and the other EfficientNet as the backbone model. PIM utilizes each pixel of an image as an independent feature, and these pixels are used as the inputs of the backbone blocks. The detailed structure of PIM is described in the original article [11]. The output of each model was a 1 × 5 vector that comprised weighted values per each class in which a higher value implies higher probability of a specific class. After training each of the four different PIMs, a Softmax function was additionally utilized to normalize the vector so that the sum of the values of the vector was 1. The Softmax function was not applied during each model (PIM) training but just afterwards. This additional process was performed before ensembling so that each PIM model could have equal contribution. Subsequently, weighted averages of the four models were used because the simple arithmetic sum of the four models seemed to overestimate the KLG in the validation set. After several trials, 2 was assigned for the two lower-grade-labeled models, while 1 was assigned for the two upper-grade-labeled models. Then, an arithmetic weighted sum average of four different vectors from the models was calculated as the final output. The class with the highest value in this average vector was selected as the class and compared with the ground-truth KLG, ranging from grade 0 to 4. The overall architecture of this model is depicted in Fig. 2, and the results of the model were augmented with Eigen-CAM [22] for visual explanations. Additionally, the computational complexity of the model was calculated using floating point operations (FLOPs) [23].

## Results

The distribution of KL grades for training, validation, and test sets is presented in Table 1.

**Table 1:** Data labeling method for each Kellgren-Lawrence grade in the study

Dataset (total number of images)	KL grade	Number of images (proportion, %)
Training set (37,462)	0	14,906 (39.8)
	1	7022 (18.7)
	2	9149 (24.4)
	3	4920 (13.1)
	4	1465 (3.9)
Validation set (9186)	0	3483 (37.9)
	1	1785 (19.4)
	2	2257 (24.6)
	3	1292 (14.1)
	4	396 (4.3)
Test set (17,040)	0	7148 (41.9)
	1	2689 (15.8)
	2	3024 (17.7)
	3	2922 (17.1)
	4	1257 (7.4)

KL, Kellgren–Lawrence

**Fig. 3:**
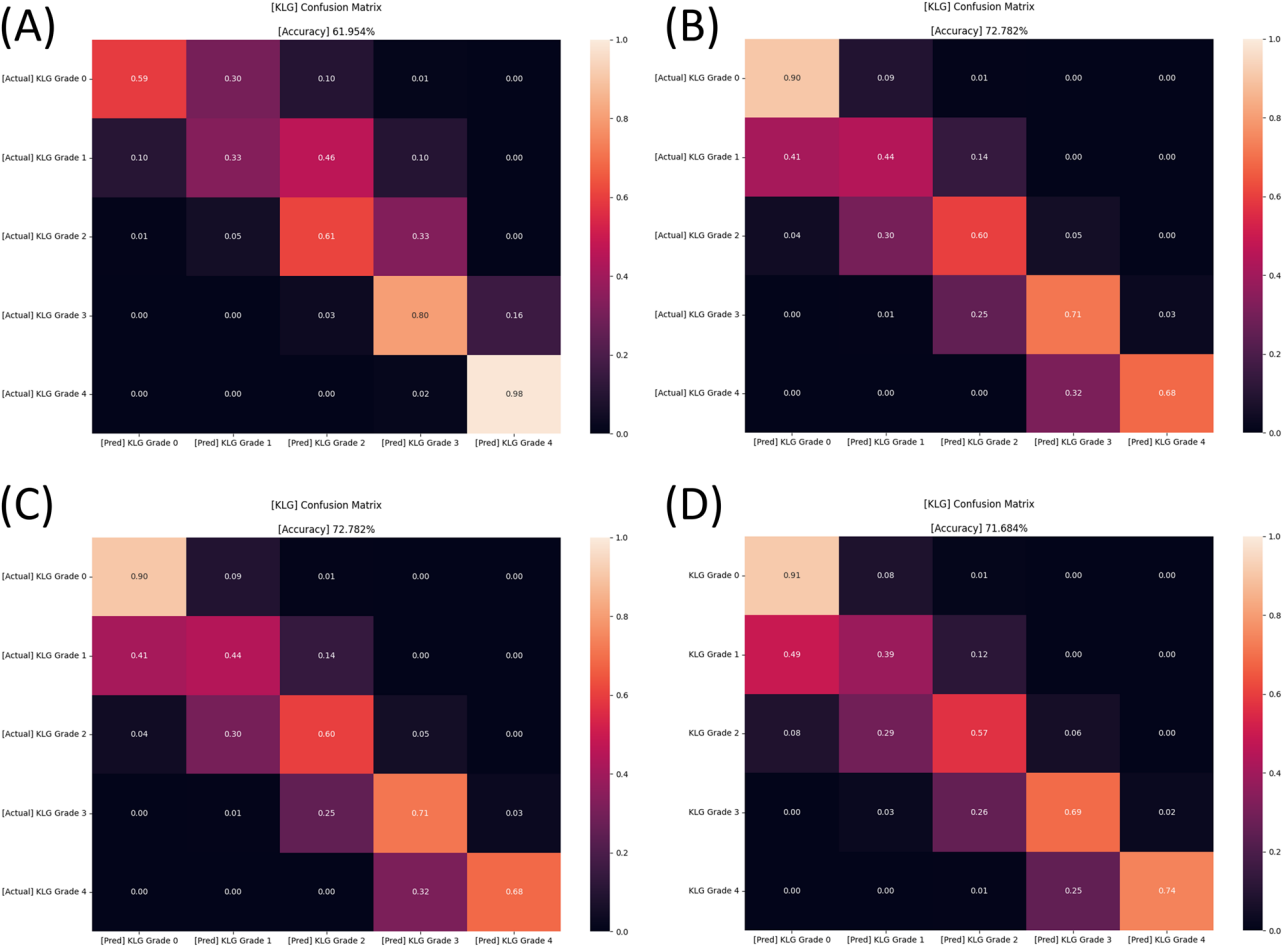
Confusion matrices of four different models (before ensemble) in the study. A PIM that applied EfficientNet and upper-grade labeling (**A**), a PIM that applied EfficientNet and lower-grade labeling (**B**), a PIM that applied Swin and upper-grade labeling (**C**), and a PIM that applied Swin and lower-grade labeling (**D**). PIM, plug-in module

**Fig. 4:**
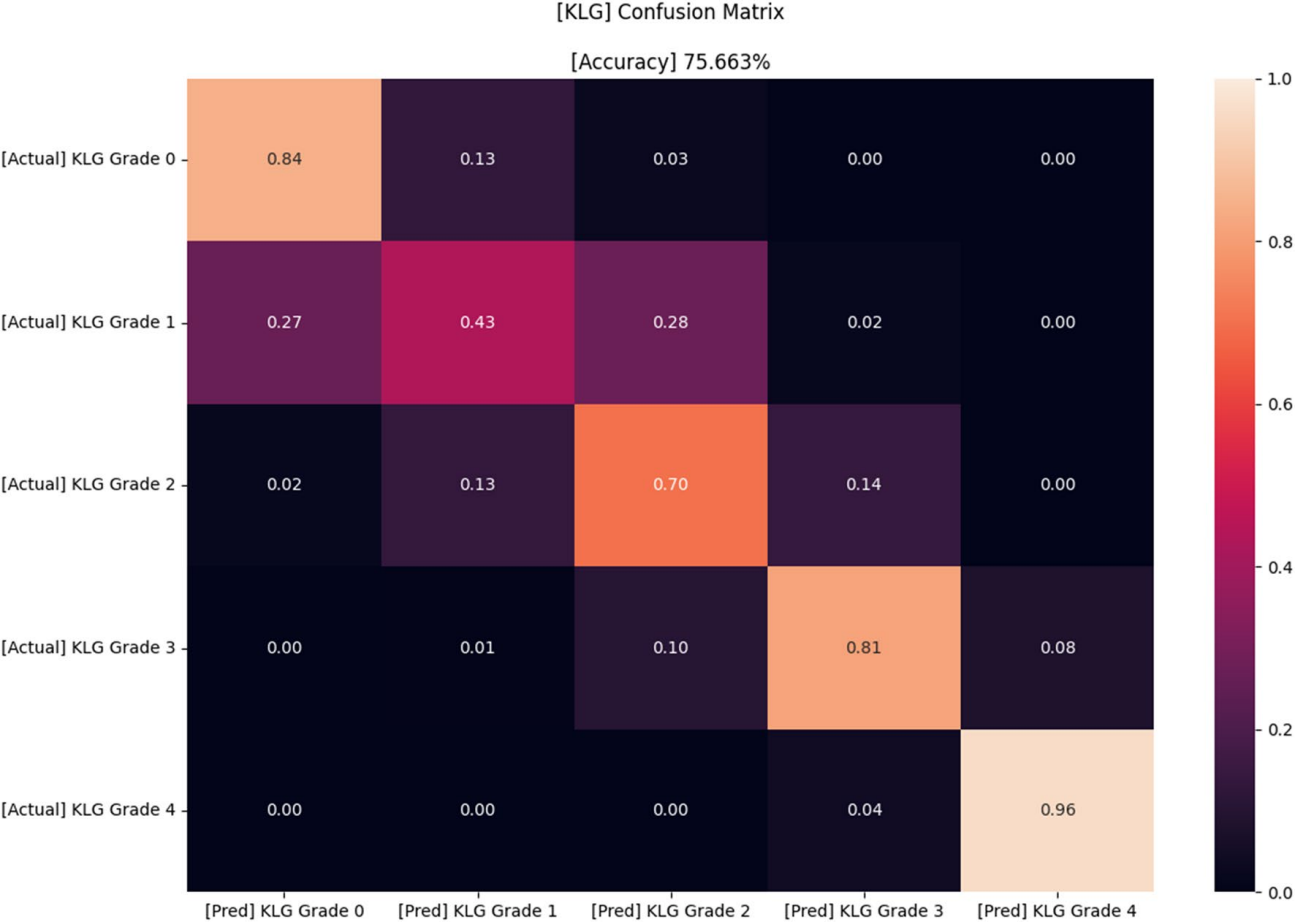
Confusion matrix of the proposed model in this study

**Fig. 5:**
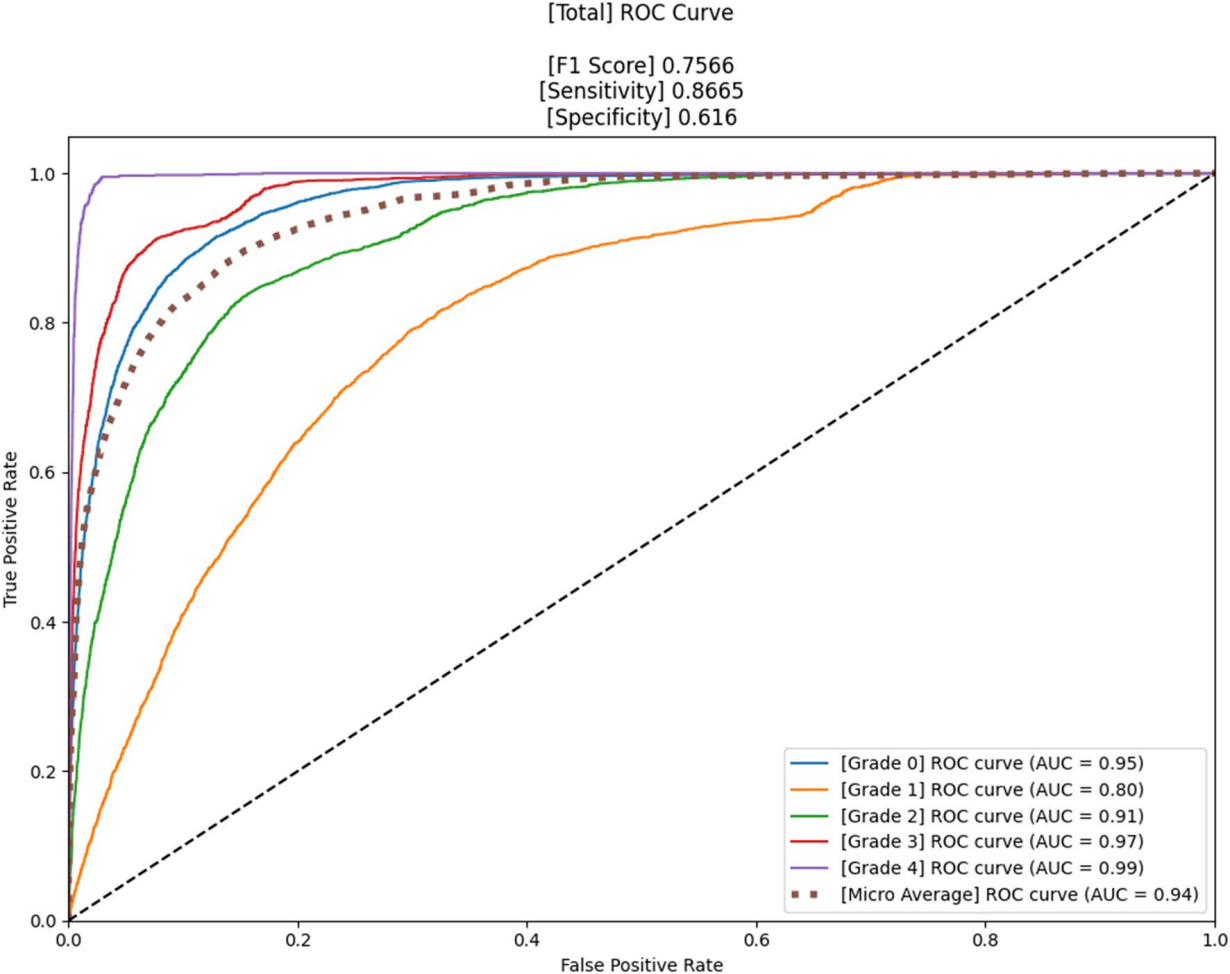
The receiver operating characteristic (ROC) curve of the proposed model in this study

The confusion matrices of four different models that were used are shown in Fig. 3, and the accuracy was 62.0%, 71.7%, 63.6%, and 72.8%, respectively. Figures 4 and 5 present the confusion matrix and the receiver operating characteristic (ROC) curve of the ensemble DL model, respectively. The overall accuracy of the model was 75.7%, and the sensitivity and specificity for each KL grade are shown in Table 2. The accuracy was the lowest for KL grade 1 (43%) and the highest for KL grade 4 (96%). The FLOPs of the ensemble model were 565.34 G.

**Table 2:** Sensitivity and specificity of the proposed model for each Kellgren–Lawrence grade

KL grade	Sensitivity	Specificity
Grade 0	0.84	0.93
Grade 1	0.43	0.93
Grade 2	0.71	0.90
Grade 3	0.81	0.95
Grade 4	0.96	0.98

KL, Kellgren–Lawrence

### Prediction visual explanations

**Fig. 6:**
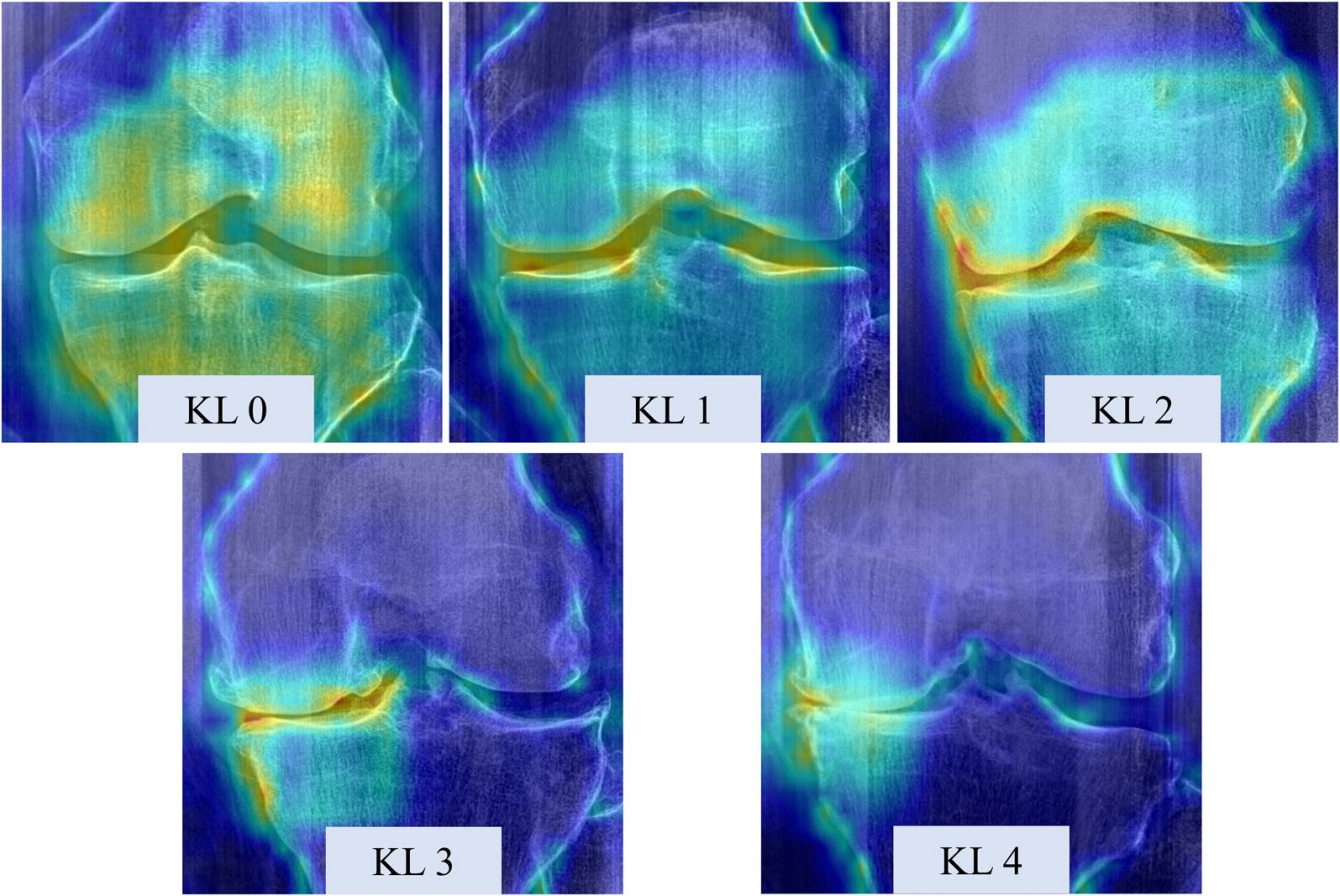
Samples of the model visual explanation using Eigen-CAM for different knee osteoarthritis severity; KL, Kellgren–Lawrence

The visual explanations of the ensemble DL model for different grades of KL are shown in Fig. 6. We could identify different patterns across different class levels of KOA and the relevant features (represented as brighter uptake in the image) matched the expected KOA features (joint space narrowing and osteophytes) in the joint margins.

## Discussion

The ensemble DL model of this study could predict KL grades with higher accuracy than most of the models that had been published previously (Table 3). Models with a minimum test set size of 500 were exclusively selected for appropriate comparison. Although the model still showed relatively lower accuracy on KLGs 1 and 2, this was superior to most of the previously proposed models. It is also noteworthy that the visual explanations of the ensemble model were in accordance with the relevant features of the model, further reinforcing the validity of the model.

**Table 3:** Comparison with state-of-the-art DL-based methods (with a minimum test set size of 500) for a KOA severity assessment task

Year	Method	DL algorithm	Test set size	Accuracy for KLG 0 (%)	Accuracy for KLG 1 (%)	Accuracy for KLG 2 (%)	Accuracy for KLG 3 (%)	Accuracy for KLG 4 (%)	Average class accuracy (%)
2016	Reference [24]	CNN	2686	71	20	56	76	80	**59.60**
2017	Reference [25]	CNN	4400	86.9	6.0	60.2	73.0	78.1	**62.29**
2018	Reference [26]	Deep Siamese CNN	5960	78	45	52	70	88	**66.70**
2019	Reference [27]	CNN ensemble	1890	Not available					**69.50**
2019	Reference [28]	CNN	1495	Not available					**64.3**
2019	Reference [5]	Modified CNN	1385	89.8	55.6	82.6	36.0	100.0	**74.3**
2019	Reference [10]	CNN ensemble	5941	83.7	70.2		68.9	86.0	**78.4**
2020	Reference [29]	CNN	1175	79	52	58	59	85	**66.0**
2020	Reference [3]	CNN ensemble	7599	94	61	90	96	97	**87.0**
2020	Reference [6]	CNN ensemble	11,743	63.0	11.0	79.8	84.8	94.9	**68.0**
2020	Reference [4]	CNN	4090	86.5	27.0	66.8	80.9	85.8	**71.0**
2022	Reference [7]	ResNet-50 + AlexNet + TL	634	99.8	99.4	99.5	99.6	99.6	**98.9**
2024	Ours	PIM ensemble	17,040	85	46	69	82	93	**75.7**

The average class accuracy was highlighted in bold

DL, deep learning; KLG, Kellgren–Lawrence grade; CNN, convolutional neural network; TL, transfer learning; PIM, plug-in module

There are several reasons why our model was successful in classifying the severity of KOA in a knee image. The most important basis of our model development was that classifying KOA severity using KLG is a fine-grained classification tasks. The previous 14 models [3–10, 24–29] were all CNN-based and have been successful in discerning KLGs 3 or 4 since these grades have distinct features: joint space narrowing. However, these previous models showed relatively low accuracy in lower grades (KLGs 0, 1, and 2). Therefore, in the current study, the ensemble of the PIM method was applied for this fine-grained classification task, and it improved accuracy in the lower grades, although discerning between KLGs 1 and 2 still has some room for improvement. The overall accuracy of KLG prediction in a recent model by Thomas et al. [4] was 71.0%. However, the accuracy for KLGs 1 and 2 was 27% and 66.8%, respectively, whereas in our study, the accuracy was significantly higher in comparison, with an accuracy of 43% and 70%, respectively. Applying vectors in KLG labeling is another unique feature that has not been proposed in the previous literature, in which scalar values were assigned for each KL grade. The authors believe that applying a vector instead of simple numbers may also have contributed to the improved accuracy since KLG is not a simple classification task but rather a classification system that becomes higher with increased KOA severity.

Swin and EfficientNet were each used as the backbones because the former transformer-based model learns using the general aspect of an image, while the latter CNN-based model learns using the local aspect of an image. The authors hypothesized that the combination or an ensemble of these two different characteristic models could lead to better predictive outcomes. In addition, by applying the upper- and lower-grade-labeling methods, the model was able to calibrate minor differences in KL grading because, in a real clinical situation, even experts in this field sometimes overestimate or underestimate KLGs. The original label smoothing method normalizes the probability of the labels to 1. However, instead of normalizing the probabilities so that the sum of the values of the vector becomes 1, the authors maintained the original KLG as 1 in the label. By utilizing this method, because a regression-type loss function was used for our model training, when the model correctly predicts the KLG, the value of the loss function becomes 0. On the contrary, if a normalized label is used (e.g., [0, 0.8, 0.2, 0, 0]), the value of the loss function is not 0, even when the model correctly predicts the KLG. Therefore, since every DL model tries to minimize the loss function, the model tends to deviate from the correctly predicted label when a normalized label is utilized. For this reason, the authors maintained the label of the original KLG as 1. As a matter of fact, our experiments showed that the results of our labeling method were better than when the normalized labels were used. Possibly as a result of this “upper- and lower-grade labeling,” our model was quite well balanced in predicting KLGs 2 and 3. For KLG 2, underestimation occurred in 15%, while overestimation occurred in 14% (Fig. 4). For KLG 3, underestimation occurred in 11%, while overestimation occurred in 8%. This was in contrast with a previous model by Thomas et al. [4] that showed similar overall accuracy in KLGs 2 and 3 to our model. In this previous model [4], underestimation occurred in 26%, while overestimation occurred in 7% in KLG 2, and underestimation occurred in 15%, while overestimation occurred in 4% in KLG 3.

Three previous models [3, 7, 10] showed superior accuracy: 87%, 98.9%, and 78.4%, respectively; however, our model was unique in that robust data from two different large cohorts, OAI and MOST (test set size of 17,040), were used. Although the average accuracy was lower than the model reported by Muhammad et al. [3], the training, validation, and test set size were significantly larger, and the test set was independent from the training set in our model. The evaluation of model accuracy on an independent dataset may have caused lower accuracy; however, because of this independent testing, our model is a more generalized model for future usage. Furthermore, using fewer ensemble base models (four in our model versus six in the previous model [3]) may lead to faster computing and subsequently better efficiency in clinical usage. A CNN-based model proposed by Abdullah et al. [7] reported the highest accuracy (98.9%) in classifying KLG, but the model was tested on a relatively small dataset (634 test images). Another CNN-based model proposed by Norman et al. [10] was tested on a relatively large dataset (5941 images) with a high average accuracy; however, the model did not discriminate between KLGs 0 and 1.

Comparing the accuracy of four individual models and the accuracy of the ensemble model, the ensemble method significantly increased the accuracy of the model by more than 3 percentage points when compared with each base model. Although this method improved the accuracy of KLG prediction, due to the usage of four different models in classifying an image, the computing time also doubled as a trade-off. Model training took about 4 full days with the state-of-the-art graphics processing unit (GPU; GeForce RTX 3090; NVIDIA, CA, USA) that was utilized in the study; however, classifying a single knee image takes a much shorter time in our model. Considering the fact that modern central processing unit (CPU) of a computer can perform around 100–200 GFLOPs (Giga FLOPs) per second, our model (565.34 GFLOPs) would take about 3–6 seconds to classify an image. Thus, our automated knee radiograph classification model can be useful in clinical practice by automatically providing the KLG of a knee image without any significant delay. Further investigations to lighten the model and reduce the computing time are needed in the future for better accessibility. High-temperature refinement and background suppression (HERBS) [30], which has been recently proposed for fine-grained classification tasks, outperformed PIM and thus could be an alternative to lighten and improve the accuracy.

There are several limitations in this study. First, our model provided a lower performance in classifying KLGs 1 and 2 compared with other grades, although it was higher than most of the previously reported literature. Second, although the KLGs of the image datasets (OAI and MOST) that were used in the study were labeled by thorough assessment and consensus by several radiologists, KLG itself can be variable even among experts due to its qualitative definition system. This may be the part of the reason why the automated classification models (including ours) show low accuracy on the lower grades. Third, overall accuracy of our model was 76.0%, which may raise concerns in clinical usage. However, the accuracy was generally high except for with grades 1 and 2; also, discerning between grade 1 and 2 actually does not critically alter decision-making in real practice. Therefore, the authors believe that the model can be readily used in the clinics, although there is much room for improvement. Last but most importantly, the model entirely relies on radiographs and does not synthesize other clinical records, such as pain or patient function. Future KOA severity grading should account for modalities other than simple radiographs to design an end-to-end model that could reflect the future prognosis of KOA. This newly proposed model would be more useful in the clinical settings than current radiograph-based models since the model directly reflects the prognosis of KOA, rather than inferring from radiographic severity.

## Conclusions

The ensemble of PIMs classified KOA severity using simple radiographs with fine accuracy. Although improvements will be needed in the future, the model has been proven to have the potential to be clinically useful.

## Data Availability

The datasets used in the study are public-use datasets. The details about acquisition of these dataset are provided at "https://nda.nih.gov/oai" and "https://most.ucsf.edu/multicenter-osteoarthritis-study-most-public-data-sharing", respectively.

## Abbreviations

KOA: Knee osteoarthritis
MRI: Magnetic resonance imaging
KLG: Kellgren−Lawrence grade
CNN: Convolutional neural network
PIM: Plug-in module
DL: Deep learning
OAI: Osteoarthritis Initiative
MOST: Multicenter Osteoarthritis Study
ROI: Range of interest
KL: Kellgren–Lawrence
FLOP: Floating point operations
ROC: Receiver operating characteristic
