# Correction: Accurate, automated classification of radiographic knee osteoarthritis severity using a novel method of deep learning: Plug‑in modules

**DOI:** 10.1186/s43019-025-00268-3

**Published:** 2025-04-29

**Authors:** Do Weon Lee, Dae Seok Song, Hyuk‑Soo Han, Du Hyun Ro

**Affiliations:** 1 Department of Orthopedic Surgery, Seoul National University College of Medicine, Seoul, South Korea; 2 CONNECTEVE Co., Ltd, Seoul, South Korea; 3 Department of Orthopedic Surgery, Seoul National University Hospital, 101 Daehak‑ro, Jongno‑gu, Seoul, 110‑744 South Korea; 4 Innovative Medical Technology Research Institute, Seoul National University Hospital, Seoul, South Korea; 5 Department of Orthopedic Surgery, Dongguk University Ilsan Hospital, Goyang, South Korea

**Correction: Knee Surgery & Related Research (2024) 36:24** 10.1186/s43019-024-00228-3

Following publication of the original article [1], we have been notified that body text contained incorrectly published parts.

The original text was as follows:

**Results**

The accuracy was the lowest for KL grade 1 (46%) and the highest for KL grade 4 (93%).

**Table 2**

**Figure:**
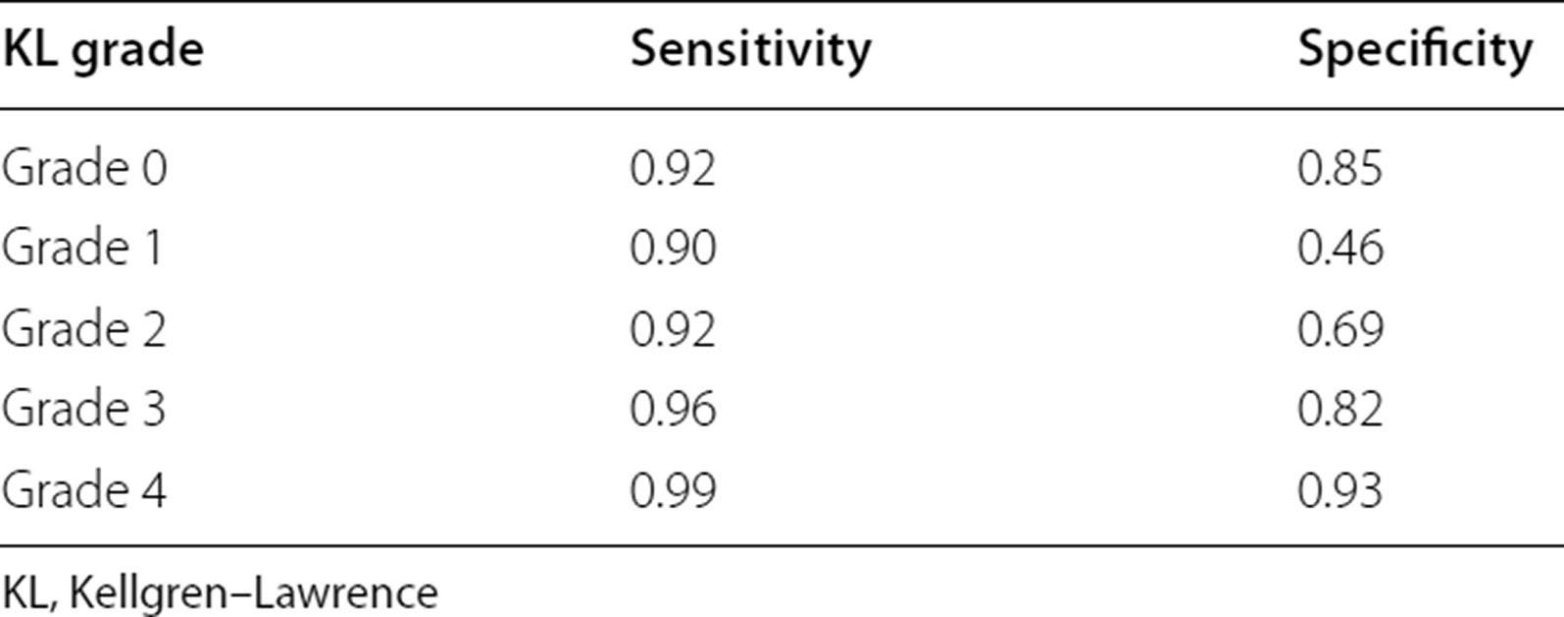
**Table 2** Sensitivity and specificity of the proposed model for each Kellgren–Lawrence grade

This has been corrected to:

**Results**

The accuracy was the lowest for KL grade 1 (43%) and the highest for KL grade 4 (96%).

**Table** 2

**Table 2:**
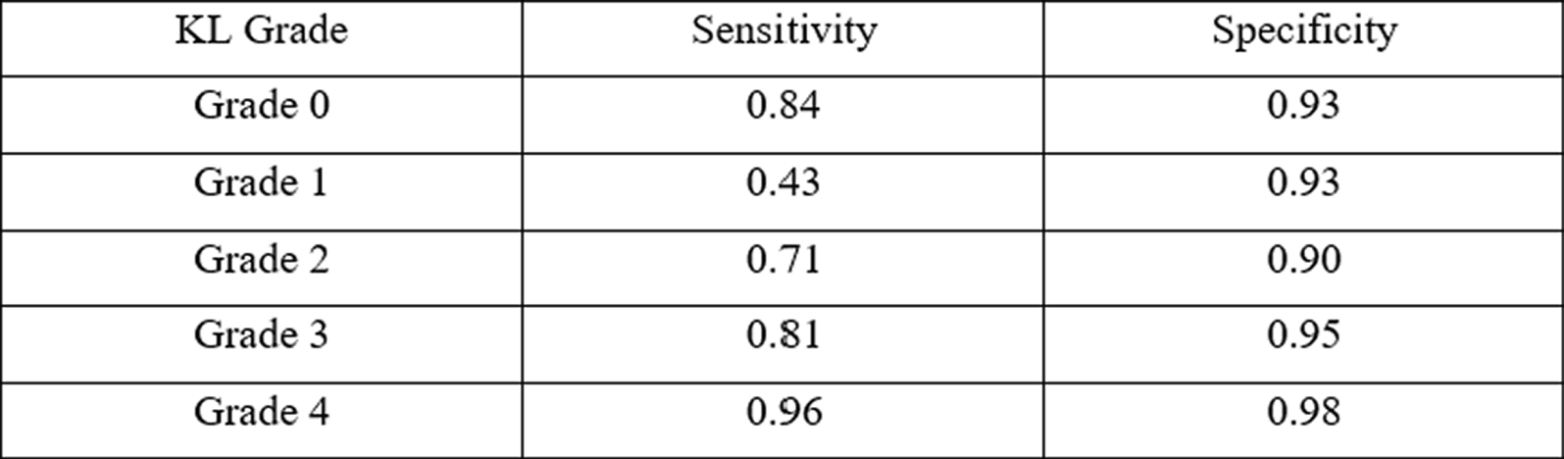
Sensitivity and specificity of the proposed model for each Kellgren–Lawrence grade

The original article was updated.
